# Effect of training on the use of long-lasting insecticide-treated bed nets on the burden of malaria among vulnerable groups, south-west Ethiopia: baseline results of a cluster randomized trial

**DOI:** 10.1186/1475-2875-9-121

**Published:** 2010-05-10

**Authors:** Amare Deribew, Fessehaye Alemseged, Zewdie Birhanu, Lelisa Sena, Ayalew Tegegn, Ahmed Zeynudin, Tariku Dejene, Morankar Sudhakar, Nasir Abdo, Fasil Tessema

**Affiliations:** 1 Department of Epidemiology, Jimma University, Jimma, Ethiopia; 2 Department of Health Education and Behavioral Sciences, Jimma University, Jimma, Ethiopia; 3 Department of Medical Laboratory and Pathology, Jimma University, Jimma, Ethiopia; 4 Oromiya Zonal health Department, Jimma, Ethiopia

## Abstract

**Background:**

In Ethiopia, the utilization of long-lasting insecticide-treated bed nets (LLITN) is hampered by behavioural factors such as low awareness and negative attitude of the community. The aim of this study was to present the design and baseline results of a cluster randomized trial on the effect of training of household heads on the use of LLITN.

**Methods:**

This baseline survey was undertaken from February to March, 2009 as part of a randomized cluster trial. A total of 11 intervention and 11 control *Gots *(villages) were included in the Gilgel Gibe Field Research Centre, south-west Ethiopia. House to house visit was done in 4135 households to collect information about the use of LLITN and socio-demographic variables. For the diagnosis of malaria and anaemia, blood samples were collected from 2410 under-five children and 242 pregnant women.

**Results:**

One fourth of the households in the intervention and control *Gots *had functional LLITN. Only 30% of the observed LLITN in the intervention and 28% in the control *Gots *were hanged properly. Adults were more likely to utilize LLITN than under-five children in the control and intervention *Gots*. The prevalence of malaria in under-five children in the intervention and control *Gots *was 10.5% and 8.3% respectively. The intervention and control *Gots *had no significant difference concerning the prevalence of malaria in under-five children, [OR = 1.28, (95%CI: 0.97, 1.69)]. Eight (6.1%) pregnant women in the intervention and eight (7.2%) in the control *Gots *were positive for malaria (P = 0.9). Children in the intervention *Gots *were less likely to have anaemia than children in the control *Gots*, [OR = 0.75, (95%CI: 0.62, 0.85)].

**Conclusion:**

The availability and utilization of LLITN was low in the study area. The prevalence of malaria and anaemia was high. Intervention strategies of malaria should focus on high risk population and vulnerable groups.

## Background

The African region south of the Sahara is heavily affected by malaria. Ethiopia is among the 30 high burden countries in malaria infection and contributed to 6% of the malaria cases in Africa. Due to climatic changes and geographic factors in Ethiopia, malaria occurs everywhere except the central high lands[1].

The use of LLITN is one of the major components of the selective vector control strategy in Ethiopia. LLITN distribution in Ethiopia primarily focuses on households with children less than five years of age and pregnant women in targeted areas[2]. The national malaria control programme had distributed 20 million LLITN between 2005 and 2007, free-of-charge, to households with vulnerable groups [1,2].

Low awareness about malaria and the utilization of the preventive methods are the serious challenges of the malaria control programmes in Africa and Ethiopia[3-7]. The level of knowledge and the use of LLITN in Ethiopia is very low[4,8]. According to the reports of the 2005 Ethiopian Demographic and Health Survey (EDHS), only 2% of rural under-five children and 1% of pregnant women used LLITN[8]. In Gilgel Gibe Field Research Centre, south-west Ethiopia, only 25% of under-five children slept under LLITN the night before the survey[9]. It was observed that many mothers in the study area had used LLITN for scarves and bed sheets to prevent louses and fleas[9]. Utilization of LLITN is also hampered by the low health service coverage, especially in the vast majority of the rural communities[8]. Cognizant of the above facts, effective utilization of LLITN in the community is unlikely in the near future. Therefore, there should be alternative strategy that empowers the community for effective utilization of LLITN to control the burden of malaria in the population particularly in the vulnerable groups (under-five children and pregnant women).

The objective of the trial is to assess the effect of tailored training of the heads of the households on the use of LLITN and community network system on the burden of malaria in vulnerable groups.

### Hypothesis of the trial

Training of the heads of the households by village residents on the proper use of LLITN will reduce the burden of malaria in under-five children and pregnant women.

The idea of educating heads of households (decision makers of the household matters) about the proper use of LLITN and assessing its impact on the burden of malaria is a new idea. Previous trials did not address the human behavior concerning the use of LLITN and its impact on the burden of malaria and anaemia among vulnerable groups [10-16]. In this article, the design and baseline results of a cluster randomized trial are presented.

## Methods

### Study area

The study was conducted in Gilgel Gibe Field Research Centre (GGFRC). This research site is selected since malaria is the major health problem in the area due to ecological disruption[17]. GGFRC is located 260 K.m south-west of Addis Ababa, the capital of Ethiopia. It was established in 2005 to serve as Demographic Surveillance System and field attachment site of Jimma University. The research centre comprised of eight rural and two urban *Kebeles *(lowest administration unit in Ethiopia) which are located around the reservoir of Gilgel Gibe hydroelectric dam. In the ten Kebeles, there are 52 *Gots*, 50,000 population and 10500 households. A *Got *is a village of 140 to 180 households within a Kebele and one Kebele may have five to seven *Gots*.

### Study design and sampling

**Figure 1:**
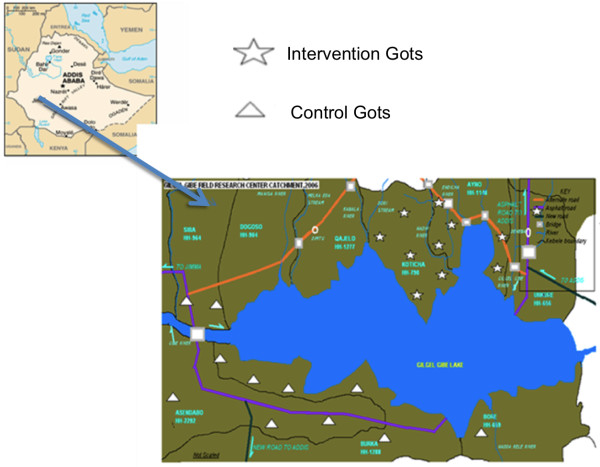
**Map of the study *Gots *and Gilgel Gibe Field Research Centre**.

As part of a cluster randomized trial, a baseline survey was conducted in GGFRC from February to March, 2009. The study population consisted of heads of households, under-five children and pregnant women in twenty-two *Gots*. Heads of the households were selected to be trained on the proper use of LLITN. The major rationale of selecting heads of the households is that they are the decision makers in all matters (including health issues) within a household. In Ethiopian, 80% of the heads of the households in rural areas are males. However, females and children are the direct beneficiaries of this intervention. To avoid contamination, villages in the field research centre were stratified into North and South of the reservoir of the dam (Figure 1).

**Figure 2:**
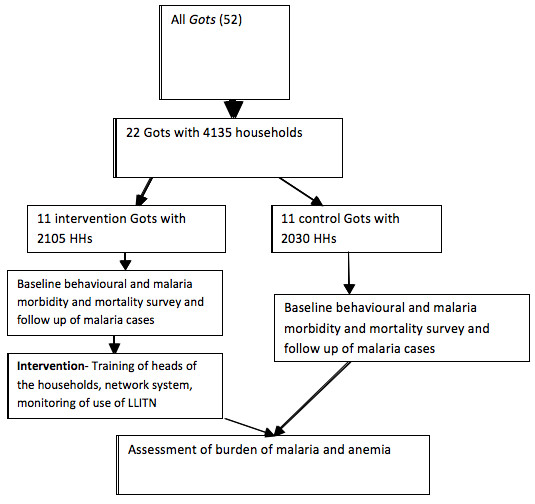
**Trial Profile, Gilgel Gibe Field Research Centre**.

*Gots *in the north direction of the reservoir were selected randomly as intervention whereas those villages in the south direction of the dam were selected to be control groups and both groups are within the same distance (10-15 Km) from the reservoir. For this trail, 22 *Gots *(11 intervention and 11 control villages) were included. Since the unit of intervention and randomization are *Gots*, the number of *Gots *that need to be included in each group is determined using the following equation[18]:

Where C is the number of *Gots*; Zα_/2 _the standard normal variable at α = 5%; Z_β _is (1-β) = 80%; P_0 _is the prevalence of LLITN utilization (27%)[9], a variable which gives maximum sample size; P_1 _is the prevalence of LLITN utilization in the intervention *Gots *(assumed to be increased by 40%); n is number of households which have vulnerable under-five children in the sampled *Gots *(n = 87); k is coefficient of variation of proportions of the outcome variable between the clusters within each group. Since there is no study to estimate K, it was taken as 0.25[18]. Based on the above information, the total number of cluster/*Gots *would be 22. All households in each cluster/*Gots *were included in the study. Detail description of the study profile is given below (Figure 2).

### The intervention and data collection procedures

#### The Intervention

The study has three phases: preparatory, intervention and evaluation phases. Baseline survey was undertaken from February to March, 2009. After the baseline survey, the intervention phase was started in May 2009 and will continue for two years. The intervention consists of tailored training of heads of the households on the proper use of LLITN and establishing community network system. Nine village residents were identified and given training of trainers (TOT) about the proper utilization of LLITN for five days. The village residents gave training to all heads of the households in each *Gots *using posters and manuals prepared in local language. After a day of the training, demonstration on use of LLITN was undertaken in the rural houses in the study villages. If the heads of the household were absent, they were trained in another days. The house level training was supervised by malaria experts from the district health offices and the investigators. Community network system was also established in the intervention villages to make the intervention sustainable. The network system links the households and the malaria control programme in the district through the trained village residents. At district level, one focal person from the malaria control team was assigned to monitor and supervise the activities of the trained village residents. The trained village residents had also parallel communication with health extension workers (HEW) who are assigned in each Kebele to give minimum health packages to each household. The village residents report their activities to the malaria control programme and HEW regularly. The investigators, the malaria control team, the trained village residents and the HEW have regular monthly monitoring meeting to discuss progress of activities and challenges.

After the training, 12500 LLITN were distributed to the control and intervention *Gots*. As of May, 2009, each household in the intervention villages has been visited by the trained village residents monthly to check for the proper use of LLITN and the occurrences of malaria. The proper use of LLITN by under-five children and pregnant women has been evaluated using observation and checklist. The proper hanging of the net in the four angles of the bed/mattress using locally made wooden materials, status of the net (new or old), and the proper placement of the net under the mattress or bed have been checked in each visit. The trained village residents also collect information on the occurrence of malaria using questionnaire.

Mass blood examination of under-five children and pregnant women has been undertaken three times a year in both the intervention and control villages to assess the burden of malaria and anaemia. In the control villages, the occurrence of malaria in each household has been monitored monthly by trained village residents. These trained residents do not give training about LLITN unlike the intervention villages.

#### Data collection procedure of the baseline survey

Socio-demographic characteristics of the study population, knowledge on malaria, availability and utilization of LLITN was assessed using structured questionnaire prepared in local language. Heads of the households who mentioned at least one symptom, one preventive method and a mosquito as a transmission agent of malaria were categorized as having good knowledge. Utilization of LLITN the night before the survey was assessed by asking the individuals whether they had slept under the net or not. Since there was no observation of the use of bed net during night, the assessment was referred as perceived utilization. However, observations were made in 220 houses (10 in each *Gots*) to check for the functionality of the net and the place where it was hanged during the survey. LLITN which had no holes and less than 3 years old were labelled as functional. The data was collected by trained high school complete individuals. Four focus group discussions were conducted by public health experts among heads of the household and spouses to explore the barriers of LLITN use.

The baseline blood investigation for all under-five children and pregnant women was done in the study *Gots*. For malaria parasite identification, two thick and thin films were prepared from a finger prick in the field and stained with Giemsa in Jimma Specialized Hospital. Each slide was read by experienced laboratory technicians. Absence of malaria parasite in 200 high power ocular fields of the thick film was considered as negative[11]. For positive cases, type of species, degree of parasitaemia and presence of gametocytes were reported. A crosscheck reading of all positives and 10% of negatives was conducted to estimate the quality of the first reading. From the positive and negative slides, a discordant rate of 2% and 4% were observed. Slides with discordant results were reread by a third microscopist. Clinical malaria was defined as the presence of malaria parasite in the blood film examination and fever[11].

Haemoglobin concentration was determined using Hemo Cue analyzer in the field *(HemoCue Hb 301, Sweden)*. Moderate and severe anaemia was defined as an haemoglobin level 7-10.9 g/dl and <7 g/dl respectively. Malarial anaemia was defined as presence of anaemia and malaria parasite at the same time[11].

### Data management and statistical analysis

Data were entered into computer, edited, cleaned, and analyzed using SPSS-16 and Epi Info 3.5.1 (Centre for Disease Control and Prevention, Atlanta) statistical software. First, univariate analysis was done to describe the socio-demographic characteristics of the study participants, utilization of LLITN and prevalence of malaria and anaemia. Cochran-Mantel-Haenszel chi-square test was used to compare utilization of LLITN and proportion of children and pregnant women with malaria and anaemia in the intervention and control *Gots*. To control for the effect of confounding variables and clustering of *Gots*, a stepwise multivariate logistic regression analysis was done. The Omnibus (p < 0.05) and Hosmer-Lomeshow (p > 0.05) tests were used to assess the performance of the logistic regression model. Adjusted odds ratio (OR) and 95%CI was used to interpret the findings. All FGD interviews were transcribed by the experts immediately after the interview. The transcribed data was commented by the investigators. After several readings, key categories & themes were identified. The data was interpreted and presented verbatim.

### Ethical considerations

The proposal was approved by Jimma University and the WHO ethical committee. Written consent was obtained from caretakers of under-five children and pregnant women. Patients with anaemia and malaria were given standard [19]treatment by the health extension workers or nearby health centres.

## Results

A sample of 4135 households and 21,673 people were included in the baseline survey. Of the sampled population, 49.7% of them were females. Under-five children and pregnant women constituted 18.6% and 2% of the sampled population.

### Perceived utilization of LLITN

Seventy-seven percent of households had at least one LLITN. In the intervention villages, similar proportion of males and females perceived that they had utilized LLITN the night before the survey. More females (70%) than males (66.4%) utilized LLITN in the control *Gots *(P = 0.001). In the intervention and control *Gots*, adults were more likely to utilize LLITN than the children (P = 0.001). Interestingly, perceived utilization of LLITN in all the age groups in control villages was high as compared to intervention villages. Fifty-six percent of pregnant women and 48.5% of the non-pregnant women of reproductive age group utilized LLITN in the intervention villages (P = 0.015). However, more women in control *Gots *were using LLITN compared to the intervention *Gots *(Table 1).

**Table 1:** Perceived utilization of long-lasting insecticide-treated bed nets in Gilgel Gibe Field Research Centre

Socio-demographic characteristics	Perceived utilization of LLITN					
	
	Intervention *Gots*			Control *Gots*		
	
	Yes	No	P-Value	Yes	No	P-value
**Sex, no (%)**						
Male	2948(47.7)	3234(52.3)	0.6	3125(66.4)	1578(33.6)	0.001
Female	2977(48.1)	3214(51.5)		3211(70.4)	1351(29.6)	
**Age in years, no (%)**			0.001			0.001
<5	1232(52.1)	1132(47.9)		1269(76.0)	401(24.0)	
5-14	1430(38.8)	2251(61.2)		1699(61.4)	1068(38.6)	
15-24	898(41.4)	1272(58.6)		951(55.6)	759(44.4)	
25-34	909(54.6)	756(45.4)		854(74.2)	296(25.8)	
35-49	994(57.8)	727(42.2)		1090(80.3)	267(19.7)	
> = 50	455(59.6)	308(40.4)		474(78.0)	134(22.0)	
**Women of reproductive age, no (%)**						
Pregnant	157(56.1)	123(43.9)	0.015	121(74.7)	41(25.3)	0.2
Not pregnant	1569(48.5)	1664(51.5)		1514(70.3)	632(29.5)	

After controlling for possible confounder, adults of the age group 35-49 [OR = 1.27, (95%CI: 1.11, 1.45)] and > = 50 [OR = 1.29, (95%CI: 1.10, 1.49)] years were more likely to utilize LLITN than under-five children in the intervention villages. Adults in the age group of 35 to 49 were 1.3 times more likely to utilize LLITN than the under-five children in the control villages, [OR = 1.3, (95%CI: 1.08, 1.56)]. Females [OR = 1.2, (95%CI: 1.10, 1.32)] were more likely to utilize LLITN than males in the control villages (Table 2).

**Table 2:** Factors independently associated with perceived use of LLITN, Gilgel Gibe Field Research Centre

	Intervention *Gots*		Control *Gots*	
	
	Crude OR (95%CI)	Adjusted OR(95%CI)	Crude OR(95%CI)	Adjusted OR(95%CI)
**Age in years**				
<5	1	1	1	1
5-14	0.58(0.52,0.64)	0.58(0.52,0.64)*	0.50(0.44,0.58*)	0.50(0.44,0.58)*
15-24	0.65(0.57,0.73)	0.64(0.57,0.72)*	0.39(0.34,0.46)*	**0.39(0.34,0.47)**
25-34	1.12(0.97,1.25)	1.10(0.96,1.26)	0.91(0.77,1.09)	0.90(0.76,1.07)
35-49	1.27(1.10,1.45)	1.27(1.11,1.45)*	1.30(1.10,1.56)*	1.30(1.08,1.56)*
> = 50	1.29(1.10, 1.49)	1.29(1.10, 1.49)	1.12(0.95, 1.42)	1.17(0.96, 1.44)
**Sex**				
Males	1	1	1	1
Females	1.10(0.9,1.10)	1.1(0.96,1.12)	1.20(1.1,1.30)*	1.21(1.10,1.32)*
**Women of reproductive age**				
Non- pregnant	1		1	1
Pregnant	**1.35(1.1,1.73)**	1.23(0.95,1.57)	1.23(0.85,1.77)	1.10(0.73.1.57)

* Statistically significant (P < 0.05)

Level of education of the heads of the households and household income did not have statistically significant association with utilization of at least one LLITN in a household. In the intervention villages, utilization of at least one LLITN in a household was more common when the heads of the household is female [OR = 1.92,(95%CI:1.34,2.74)] and has good knowledge on malaria [OR1.96, (95%CI: 1.36,2.8)](Table 3).

**Table 3:** Characteristics of household heads and utilization of LLITN, Gilgel Gibe Field Research Centre

Characteristics of the heads of households	Utilization of at least one LLITN in a household					
	
	Intervention *Gots*			Control *Gots*		
	
	Yes	No	Adjusted OR (95%CI)	Yes	No	Adjusted OR (95%CI)
**Literacy status**						
Illiterate	1093(87.5)	156(12.5)		1091(91.5)	102(8.5)	1
Literate	298(90.0)	33(10.0)	1.28(0.86, 1.36)	255(91.1)	25(8.9)	1.10(0.60, 1.66)
**Sex**						
Male	632(84.8)	113(15.2)	1	517(90.5)	54(9.5)	1
Female	762(91.0)	75(9.0)	1.92(1.34,2.74)*	826(91.9)	73(8.3)	1.18(0.81,1.70)
**Monthly income of the household(in Birr, median income = 500)**						
<500	491(87.5)	70(12.5)	1	345(90.8)	35(19.2)	
> = 500	494(87.1)	73(12.5)	1.04(0.67,1.34)	560(92.9)	43(7.1)	1.32(0.82,2.10)
**Knowledge about malaria**						
Poor	968(89.5)	114(10.5)	1	387(91.7)	35(8.3)	1
Good	428(85.1)	75(14.9)	1.96(1.36,2.80)*	959(91.2)	92(8.8)	0.95(0.60, 1.51)

* Statistically significant (P < 0.05)

More than 95% of the houses were conical shapes, thatched roofs and high ceiling. Observation showed that 26(24%) of the houses in the intervention and 28(26%) in the control *Gots *had functional LLITN. As a result of the nature of the house, the community were not aware how to hang and put the nets under the bed/mattress. Only 30% of the observed LLITN in the intervention and 28% in the control *Gots *were hanged properly over the bed/mattress. It was observed that only 39% and 34% of the LLITN in the intervention and control *Gots *could reach under the mattress/bed while hanged.

The results of the focus group discussion were similar to the observations. Low awareness was one of the major reasons for the low utilization of LLITN. A 30-year old housewife stated, "*We have one net in our house, but we do not know how to hang and use it. The ceiling is too high for the net to reach." *Most of the FGD participants stated that education and training should be given to them how to utilize LLITN.

A 50-year old male head of household in the control village expressed his idea, "*Awareness creation supported by demonstration how to properly use and wash bed nets should be given to us by the government and other stakeholders*."

Lack of LLITN was the other major reason of low utilization of LLITN. It was indicated that most of the households had got only one LLITN three years back. A 45-year old female in the intervention *Gots *says, "*The government gave us only one net for a house which has 10 members. How could we share one net for ten people?"*

### Prevalence of malaria and anaemia in under-five children and pregnant women

A sample of 2750 children gave blood for the diagnosis of malaria and anaemia. However, 2410(87.6%) of them were included in this report. The rest were excluded due to missing information on main variables. The mean age of children was 33.5(SD ± 1.8) and 32.1(SD ± 1.75) months in the intervention and control *Gots *respectively. The proportion of male and female children was similar in the control and intervention *Gots *(Table 4).

**Table 4:** Socio-demographic characteristics of under-five children (n = 2410) who were investigated for malaria in Gilgel Gibe Field Research Centre

Variable	Intervention(n = 1207) No (%)	Control(n = 1203) No (%)
**Sex**		
Male	616(51.0)	607(50.5)
Female	591(49.0)	596(49.5)
**Age in months**		
<12	246(20.4)	263(21.9)
12-23	62(5.1)	94(7.8)
24-35	244(20.2)	222(18.5)
36-47	236(19.6)	226(18.8)
> = 47	419(34.7)	398(33.0)
**Mean age in months(S**D)	33.5(SD ± 1.8)	32.1(SD ± 1.75)
**Birth order**		
1-3	665(55.2)	586(48.8)
4-5	364(30.2)	396(33.0)
>5	175(14.2)	219(18.2)
Mean Weight(SD)	11.1(SD ± 3.1)	11.2(SD ± 3.1)

The prevalence of malaria in under-five children in the intervention and control *Gots *was 10.5% and 8.3% respectively. The intervention and control *Gots *had no significant difference concerning the prevalence of malaria in under-five children, [OR = 1.28, (95%CI: 0.97, 1.69)]. Fifty-nine percent of malaria cases in the intervention *Gots *and sixty percent of the malaria cases in the control *Gots *were due to *Plasmodium vivax*. Four percent and three percent of children had clinical malaria in the intervention and control *Gots *respectively. After controlling for potential confounding variables, children in the intervention *Gots *were less likely to have anaemia than children in the control *Gots*, [OR = 0.75, (95%CI: 0.62, 0.85)]. Under-five children in the intervention *Gots *were 1.36 times more likely to have fever than children in the control *Gots*, [OR = 1.36, (95%CI: 1.14, 1.63)] (Table 5). There was no significant difference of the prevalence of malaria among age groups, sex and birth order of the under-five children (Table 6).

**Table 5:** Prevalence of malaria and anaemia in under-five children in the intervention and control villages (n = 2410) in Gilgel Gibe Field Research Centre.

Variable	Intervention *Gots *No (%)	Control *Gots *No (%)	Adjusted OR(95%CI)*
**Malaria parasitaemia**			1.28(0.97,1.69)
Yes	127(10.5)	100(8.3)	
No	1080(89.5)	1103(91.7)	
**Species of malaria parasite**			1.10(0.62,1.90)
*Plasmodium falciparum*	52(40.9)	38(38.0)	
*Plasmodium vivax*	75(59.1)	60(60.0)	
**Fever**			**1.36(1.14,1.63)†**
Yes	380(31.5)	304(25.3)	
No	827(68.5)	899(74.7)	
**Clinical Malaria**			1.47(0.95,2.27)
Yes	52(4.3)	36(3.0)	
No	1155(95.7)	1167(97.0)	
**Anaemia**			**0.75(0.62,0.85)†**
Yes	352(29.2)	428(35.6)	
No	855(70.8)	775(64.4)	
**Severe Anaemia**			
Yes	12(1.0)	20(1.7)	0.58(0.28,1.20
No	1190(99.0)	1181(98.3)	
**Malarial Anaemia**			0.81(0.53,1.24)
Yes	40(3.3)	50(4.2)	
No	1159(96)	1138(94.6)	

*Adjusted for age, sex and birth order of children

†P-value < 0.05

**Table 6:** Malaria infection by demographic characteristics of the children in Gilgel Gibe Field Research Centre

Variable	Malaria parasite					P-value
		
	Intervention *Gots*			Control *Gots*		
	Yes	No		yes	No	
**Sex**						0.74
Male	73(11.9)	543(88.1)	0.12	52(8.6)	555(91.4)	
Female	54(9.1)	537(90.9)		48(8.1)	548(91.9)	
**Age in months**						0.067
<12	20(8.1)	226(91.9)	0.6	23(8.7)	240(91.3)	
12-24	7(11.3)	55(88.7)		5(5.3)	89(94.7)	
25-34	24(9.8)	220(90.2)		23(10.4)	199(89.6)	
35-47	26(11.0)	210(89.0)		26(11.5	200(88.5)	
> = 47	50(11.9)	369(88.1)		23(5.8)	375(94.2)	
**Birth order**						0.55
1-3	63(9.5)	602(90.5)	0.35	48(8.2)	538(91.8)	
4-5	45(12.4)	319(87.6)		37(9.3)	359(90.7)	
>5	19(10.9)	156(89.1)		15(6.8)	204(93.2)	

A sample of 242 pregnant women was included for the diagnosis of malaria and anaemia. The mean age of these women was 26.1(SD ± 5.7) and 26.4(SD ± 5.3) years in the intervention and control *Gots *respectively. Eight (6.1%) pregnant women in the intervention *Gots *and eight (7.2%) in the control *Gots *had malaria parasite (P = 0.9). *Plasmodium falciparum *accounted 57% and 50% of the malaria cases in pregnant women in the intervention and control *Gots *respectively. Twenty-nine percent of the pregnant women in the intervention villages and 36.0% of the women in the control villages had moderate (haemoglobin level of 7 to 11 g/dl) anaemia (P = 0.3). Three percent and five percent of the pregnant women had malarial anaemia in the intervention and control *Gots *respectively (Table 7).

**Table 7:** Prevalence of malaria and anaemia in pregnant women (n = 242), Gilgel Gibe Field Research Centre

Variable	Intervention *Gots*	Control *Gots*	P-value
Mean age in years(SD)	26.1(SD ± 5.7)	26.4(SD ± 5.3)	0.76
			
**Fever in the last two weeks, no (%)**			0.02
No	88(67.2)	90(81.1)	
Yes	43(32.8))	21(18.9)	
**Parasitaemia, no (%)**			0.9
No	123(93.9)	103(92.8)	
Yes	8(6.1)	8(7.2)	
**Anaemia, no (%)**			0. 36
No	91(70.0)	71(64.0)	
Moderate	38(29.2)	40(36.0)	
Severe	1(0.8)	0(0.0)	
**Malaria anaemia**			0.8
Yes	4(3.1)	5(4.5)	
No	127(96.9)	106(95.5)	

## Discussion

Perceived utilization of LLITN is higher in the present study as compared to the findings of other literatures[20-22]. The control villages were better in the perceived utilization of LLITN than the intervention *Gots*. However, during observation, all villages were poor in utilization of LLITN. Most rural people who lived in traditional round houses (*Tukul*) did not utilize LLITN properly. As a result of the nature of the houses (mud wall and conical roof), most people do not know how to hang the four angles of the rectangular LLITN. Most of the observed hanged nets could not reach the edge of the bed or mattress which might contribute for the high burden of malaria in the area. Lack of awareness and skill on the use of LLITN and shortage were also mentioned by most of the focus group discussants. Lack of awareness on the utilization of LLITN was also reported by Belay *et al *in north Ethiopia[21]. Educating the community to use simple locally made methods of hanging LLITN could improve the utilization of LLITN significantly. Unlike other studies, use of LLITN is not associated with the literacy status of heads of the households [21]. However, children less than the age of five years and adolescents were less advantageous than adults in the utilization of LLITN in this study.

In this study, the malaria prevalence survey was undertaken in dry (non-epidemic) season. However, the prevalence of malaria in children (10.5%) in the study area was very high compared to the findings of the national malaria indicator survey (4%)[22], Shargie (2.4%) [23]and Endeshaw et al (4.1%)[24]. The prevalence of malaria in Gilgel Gibe was 10.5% in the epidemic season three years back[17]. This indicates that the burden of malaria is still high in the area unlike the national prevalence, which has dramatically decreased over the last decade[25]. The effect of the lake created by the dam might have contributed for the high burden of malaria in the area[17]. Unlike other studies[22,23], the prevalence of *Plasmodium vivax *is higher than *Plasmodium falciparum*. Widespread use of artemether/lumefantrine by the community through the health extension workers may explain the low prevalence of *P. falciparum*[26]. Prevalence of malaria among pregnant women in this study (6.5%) was also higher compared to the previous findings (1.8%) by Newman et al[27]. *Plasmodium falciparum *was the common parasite in pregnant women, which can result in serious consequences on the outcome of pregnancy.

Anaemia (haemoglobin level < 11 g/dl) is multifactorial in origin and remain the major public health problem of children and pregnant women in Africa and Ethiopia[22,28,29]. The prevalence and causes of anaemia is not well studied in Ethiopia[30]. The prevalence of anaemia in children in this study was higher than the finding of Wolde et al in north Ethiopia [30] but comparable with the report of EDHS[8]. The prevalence of malarial anaemia in the present study is lower than the findings of other investigators elsewhere[8]. The reversal of malaria parasite prevalence from *P. falciparum *to *P. vivax *could explain the lower malarial anaemia in the present study. In major epidemic seasons, the proportion of children with malaria anaemia may be higher than the current finding in the study area.

A significant proportion of pregnant women in the control (36%) and intervention (29%) *Gots *had moderate anaemia. This finding is much higher than the reports of the EDHS (17%)[8]. In high malaria burden areas like Gilgel Gibe, pregnant mothers may have repeated attacks of malaria, which predispose them to anaemia.

Even though, we tried to document the burden of anaemia in vulnerable groups, the causes of anaemia remain unclear. The definition of malarial anaemia might not explain the real association of anaemia and malaria. Serum ferritin level was not assessed due to resource constraints.

## Conclusion

The utilization of LLITN was hampered by lack of awareness in the rural community who lives in traditional *Tukul *houses. The prevalence of malaria and anaemia in under-five children and pregnant women in the study area was high compared to the national trend of malaria. Intervention strategies of malaria and anaemia should focus on the high risk population like Gilgel Gibe Field Research centre and vulnerable groups.

## Competing interests

The authors declare that they have no competing interests.

## Authors' contributions

AD conceived the study and was involved in the design, coordination, field supervision, analysis and drafted the manuscript. FA participated in the design, field supervision and report writing. ZB was involved in field supervision and writing of the qualitative report. MS and AZ were involved in field supervision and reviewed of the article. NA mobilized the community during the mass blood examination and reviewed the article. AT and LS were involved in field supervision and report writing. FT and TA were involved in data analysis and field supervision and reviewed the article. All authors read and approved the manuscript.
